# Analytical Model for Particle Capture in Nanopores Elucidates Competition among Electrophoresis, Electroosmosis, and Dielectrophoresis

**DOI:** 10.1021/acsnano.0c06981

**Published:** 2020-11-10

**Authors:** Mauro Chinappi, Misa Yamaji, Ryuji Kawano, Fabio Cecconi

**Affiliations:** † Dipartimento di Ingegneria Industriale, Università di Roma Tor Vergata, Via del Politecnico 1, 00133 Rome, Italy; ‡ Department of Biotechnology and Life Science, Tokyo University of Agriculture and Technology, Tokyo 184-8588, Japan; § CNR-Istituto dei Sistemi Complessi, Via dei Taurini 19, I-00185 Rome, Italy

**Keywords:** nanopores, capture process, generalized Smoluchowski model, electrohydrodynamics, dielectrophoretic capture, α-hemolysin, protein sensing

## Abstract

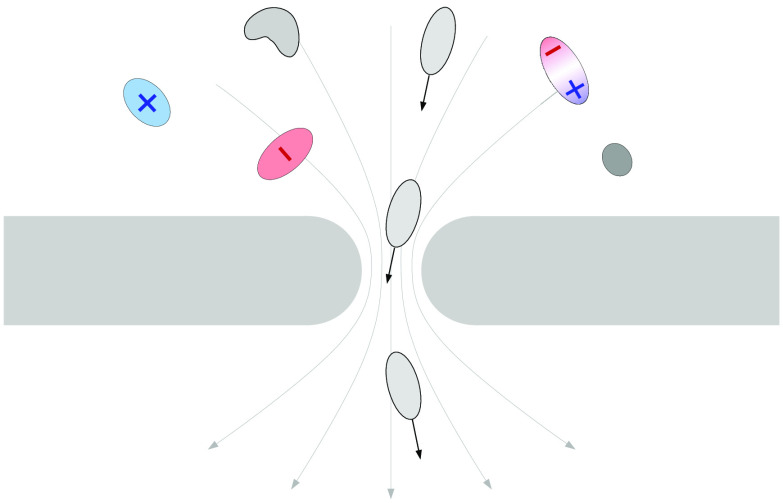

The interaction between nanoparticles dispersed in a fluid and nanopores is governed by the interplay of hydrodynamical, electrical, and chemical effects. We developed a theory for particle capture in nanopores and derived analytical expressions for the capture rate under the concurrent action of electrical forces, fluid advection, and Brownian motion. Our approach naturally splits the average capture time in two terms, an *approaching time* due to the migration of particles from the bulk to the pore mouth and an *entrance time* associated with a free-energy barrier at the pore entrance. Within this theoretical framework, we described the standard experimental condition where a particle concentration is driven into the pore by an applied voltage, with specific focus on different capture mechanisms: under pure electrophoretic force, in the presence of a competition between electrophoresis and electroosmosis, and finally under dielectrophoretic reorientation of dipolar particles. Our theory predicts that dielectrophoresis is able to induce capture for both positive and negative voltages. We performed a dedicated experiment involving a biological nanopore (α-hemolysin) and a rigid dipolar dumbbell (realized with a β-hairpin peptide) that confirms the theoretically proposed capture mechanism.

Nanopores and nanoporous membranes are ubiquitous in disruptive technologies for single molecule sensing,^1,2^ blue-energy harvesting,^3,4^ and water desalination.^5,6^ In all of these applications, the precise characterization of ion and water flows across the pore, along with the comprehension of the interaction between nanoparticles dispersed in the solution and the pore, is of enormous relevance. In nanopore sensing, molecules to be analyzed must be easily captured by the pore, whereas the access of undesired molecules must be limited. In nanopore water treatment systems and blue-energy porous membranes, dispersed nanoparticles may clog the pore (fouling^7^), dramatically reducing the performance of real-life devices with respect to the highly controlled laboratory setups.

Most of the experimental results on the nanoparticle–nanopore interaction have come from single nanopore recordings. The typical experimental setup consists of two chambers of an electrolyte cell that are connected by a single biological^8−10^ or solid-state nanopore.^11−14^ A voltage applied across the two chambers induces an ionic current. Artificial nanoparticles or biomolecules, added to one of the two chambers, interact with the pore, either bumping on its entrance or translocating across it, and hinder the passage of ions, leaving a signal in the electric current trace. In the last 25 years, mainly fostered by biomolecule-sensing applications, a large amount of literature reported experimental evidence on nanopore–nanoparticle interactions, highlighting that particle capture and transport can be achieved through a variety of different mechanisms.

Electrophoresis is, by far, the most widely explored effect due to its relevance in nanopore DNA sequencing.^15^ Moreover, electrophoresis was also employed in nanopore experiments involving peptides, proteins, or protein aggregates^16,17^ (that, in general, carry a relatively small charge) and nanoparticles that, when immersed in an electrolyte solutions, typically acquire a surface charge.^18,19^ The application of a voltage across the membrane results in an electrical field, **E** (see Figure 1a). As the membranes used in nanopore devices have a low dielectric constant compared to that in the electrolyte solution (ϵ_r_ ≃ 80 for water, ϵ_r_ ≃ 3 for lipid bilayers^20^), the electrical field funnels into the pore. Charged molecules, hence, are driven toward or away from the pore, depending on the sign of their charge. The only competing effects to this transport are the Brownian diffusion and the overcoming of a free-energy barrier at the pore entrance, with the latter being particularly relevant for polymer molecules (*e.g.*, single-strand DNA) whose capture in the pore occurs only through a large entropy reduction.^21^

**Figure 1:**
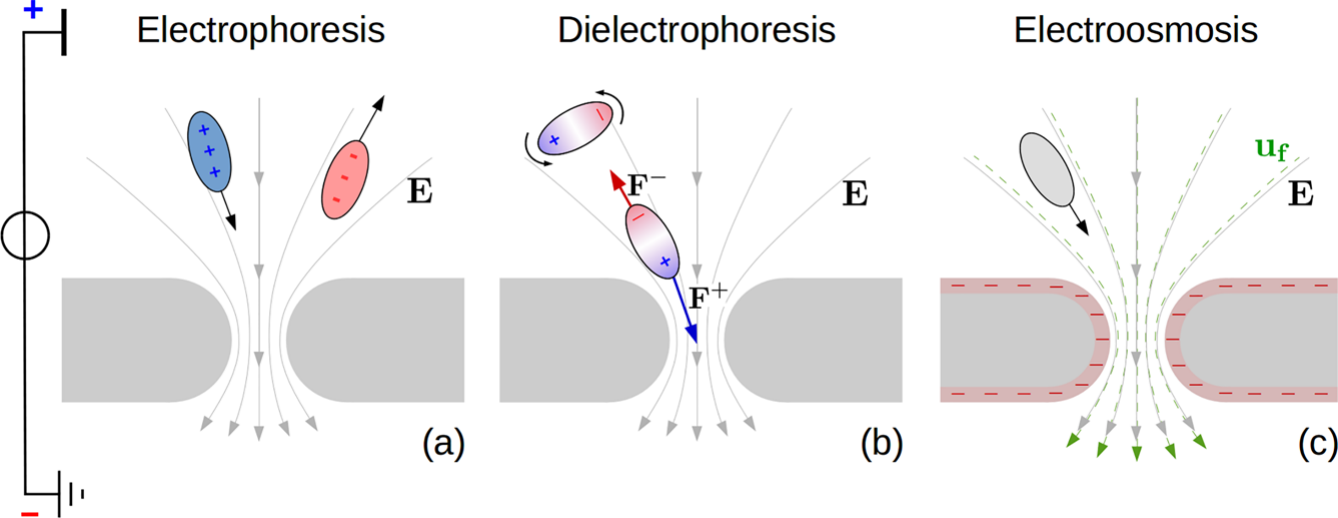
Main capture mechanisms. A voltage Δ*V* applied between the two sides of the membrane generates a funnel-shaped electrical field. Charged particles are driven toward or away from the nanopore, depending on the sign of their charge (a). Neutral particles carrying an intrinsic dipole orient themselves along the field lines. An attractive force affects the portion of the molecule closer to the pore, **F**^**+**^ in the example in panel (b), whereas a repelling force **F**^**–**^ acts on the portion far from the pore, with |**F**^**+**^| > |**F**^**–**^|. Consequently, the molecule is attracted by the nanopore. Finally, if a fluid flows across the nanopore, dispersed particles are dragged into the pore (c). In the example of panel (c), the flow is due to electroosmosis. The pore is negatively charged, so there is an accumulation of positive ions inside the pore. Consequently, a net force in the direction of the electrical field **E** acts on the fluid, generating a net velocity flow **u**_**f**_ (green dashed line). The flow can be generated also by other forces such as a pressure difference between the two sides of the membrane.

Nanoparticle or biomolecule capture can be induced also by advection. In this case, a fluid flow, directed toward the pore, drags the particles dispersed in the solution independently of their charge. The flow can be generated by a pressure difference between the two sides of the membrane^22,23^ or by electroosmosis^24^ (see Figure 1c). The latter phenomenon is quite common in nanofluidics as nanopore surfaces are usually charged. The surface charge induces an accumulation of counterions and a depletion of co-ions inside the pore. As a consequence, when the voltage is applied across the two chambers, the unbalance between positive and negative charges inside the pore results in a net force on the solvent so strong to move the fluid. Electroosmosis is hence deeply entangled with the ionic flow, as both depend on pore shapes and their charge distribution, leading to complex electrohydrodynamic patterns.^25−27^ Electroosmosis can either compete or cooperate with electrophoresis in particle capture.^28−31^ An example of competition is discussed in ref (30), where the electroosmosis was shown to induce capture of peptides against electrophoresis.

A last active mechanism affecting particle dynamics in nanopore systems is dielectrophoresis. In this work, we discuss only the cases of a stationary electric field hence, limiting our approach to direct current dielectrophoresis. Moreover, we focus on particles carrying an intrinsic dipole. Those particles orient their dipole along the electrical field **E** and move in the direction of increasing |**E**| (see Figure 1b). Experimental evidence of dielectrophoretic capture has been provided for flexible polymers,^32,33^ and a toy model for capture and trapping of a permanent dipole (nanopore tweezer) has been proposed by two of us.^34^

Finally, when the nanoparticles are distant from the nanopore just a few atomic lengths, their dynamics is also affected by the chemical interactions between the particles and the pore. These interactions, strongly dependent on the specific nature of the particles, can be exploited to favor the capture and the dwelling of the particle in the nanopore; furthermore, they can compete or cooperate with electrical effects (see, among others, refs (35−37)).

Several theoretical approaches for describing nanoparticle capture have appeared in the literature. The most promising attempts are based on a steady-state absorption problem formulated through a generalized Smoluchowski equation.^38−41^ In essence, the particle is modeled as a material point under the action of an external driving (*e.g.*, electrophoresis) and Brownian diffusion. Different versions of this approach have been proposed with the aim of reproducing specific experimental conditions. Wong and Muthukumar^39^ studied the diffusion-limited capture regime under electroosmotic flow for a charged flexible polymer (DNA). Grosberg and Rabin^38^ modeled the free-energy barrier at the pore entrance as a spatially extended repulsive potential. Although literature mainly focused on DNA,^39,42^ Smoluchowski-like approaches have been often applied to explain experimental capture rates of other molecules, such as folded proteins^43^ and nanoparticles.^44^ The Smoluchowski equation can be hence considered the reference theoretical tool to study molecule capture in confined geometries. However, most of the times, Smoluchowski-like approaches have been used to get *a posteriori* theoretical justification to explain the experimental observations.

In this work, we employ a generalized Smoluchowski equation to describe the capture of rigid nanoparticles in nanopores under the concurrent action of electrical forces, fluid advection, and Brownian motion. We find explicit criteria for *a priori* assessment of which effects, among electrophoresis, electroosmosis, and dielectrophoresis, dominate the capture. Differently from other studies, pore–particle chemical interaction is taken into account in an effective manner by a partially adsorbing boundary condition. We derive analytical expressions for the capture frequency showing that the average capture time is the sum of an *approaching time* due to the motion of the particle from the bulk to the pore mouth and of a *entrance time* determined by the presence of a free-energy barrier at the pore entrance. Our analytical results are applied to representative literature results ranging from electrophoretic capture to competition between electrophoresis and electroosmosis. Interestingly, our model predicts that dielectrophoresis is able to induce the capture of rigid particles at both positive and negative voltages. As, to the best of our knowledge, no experimental data are available in the literature on dielectrophoretic capture of rigid molecules in nanopores, we designed a dedicated experiment involving α-hemolysin^45^ (a biological nanopore widely employed in sensing experiments^9,16,28,30,32,36,46−48^) and a rigid dipolar dumbbell realized with a β-hairpin peptide. A nice agreement is found between the model prediction and the experimentally measured capture rates.

The article is structured as it follows. First, we present the generalized Smoluchowski equation and the general stationary solution we found for partial adsorbing boundary. Then, we report the analytical solution under the combined action of electrophoresis, electroosmosis, and dielectrophoresis for approaching and entrance frequencies. Finally, we apply our theory to the interpretation of experimental data reported in the literature for peptide and protein capture, and we discuss the experiment we performed for dielectrophoretic capture.

## Results and Discussion

### Capture Modeling

**Figure 2:**
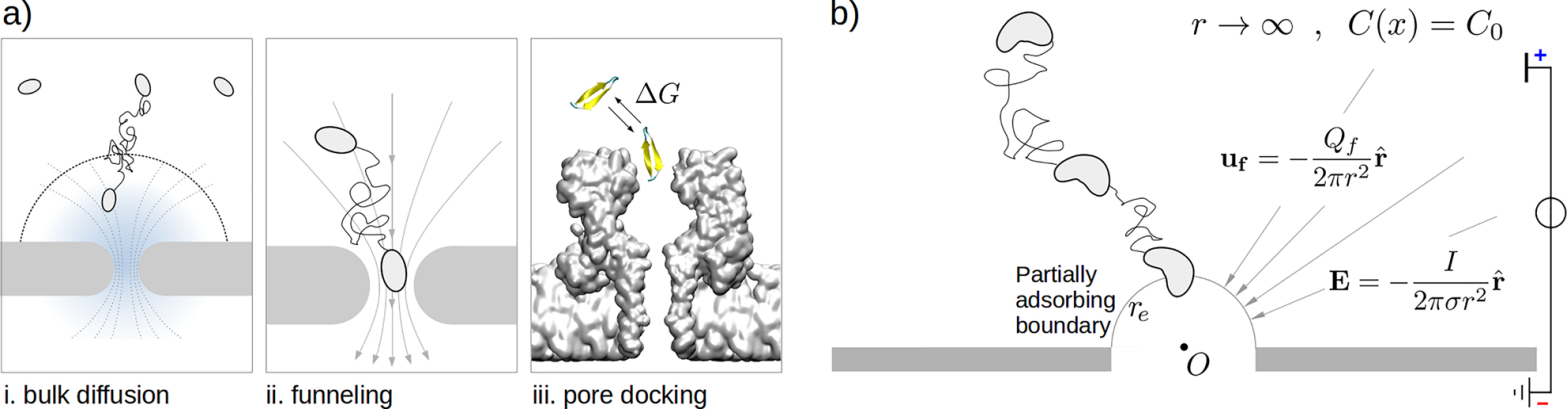
Particle capture in nanopores. (a) Three phases of the capture. (i) Bulk diffusion. Far from the pore, the forces acting on the particles (*e.g.*, electrophoresis, electroosmotic drag) are negligible as they typically scale as *r*^–2^, with the distance *r* from the pore entrance. Hence, the particle dynamics is dominated by the Brownian motion until the particle reaches the pore capture region where the diffusion and the active forces become comparable. (ii) Funneling. Once the particle is in the capture region, the Brownian motion competes with the electric and hydrodynamic forces. Supposing that the latter are directed toward the pore, the particle experiences a funnel-like force field: the closer the particle is to the pore entrance, the larger the attractive force. (iii) Pore docking. The particle finally reaches the pore entrance region, where chemical pore–particle interactions become relevant. The particle needs to overcome a free-energy barrier Δ*G* to enter the pore. Panel (iii) was realized using the VMD software.^49^ (b) Continuum model of particle capture. The advection–diffusion eq 3 is solved in the hemispherical shell between pore entrance radius *r*_e_ and the bulk radius *r*_b_ (in the following, we will report results for *r*_b_ → *∞*, so that for *r* → *∞* the concentration reaches the bulk value *C*_0_). Radial symmetry is assumed, so all the quantities depend only on the distance *r* from the origin *O*. Partially adsorbing boundary condition (Robin), eq 5, is imposed at *r*_e_. Particles are affected by a radial electrical field **E**, eq 14, and they are advected by an incompressible radial velocity field **u**_**f**_, eq 11.

The capture of a single molecule into a nanopore is a complex process governed by the interplay among Brownian diffusion, hydrodynamics, and chemical and electric effects. Each effect does not play the same role in all of the stages of the capture process. For instance, the electric field intensity and the solvent velocity induced by electroosmosis or pressure gradients decrease with the distance between the molecule and the pore entrance, whereas chemical interactions are relevant only when the molecule and pore are practically in contact. In general, we can distinguish three main stages of the particle capture (see Figure 2a). (i) Bulk diffusion: Far from the pore, the coherent forces acting on the particles (*e.g.*, electrophoresis and electroosmotic drag) are negligible. Here, the dynamics is dominated by the Brownian motion of the particle in the bulk. (ii) Funneling: The Brownian motion can bring the particle in the pore capture region. Here, Brownian diffusion competes with the electric and hydrodynamic forces. Supposing that the latter are directed toward the pore, the particle experiences an effective funnel-like force field, for which the closer the particle is to the pore, the larger the attractive force. (iii) Pore docking: The particle finally reaches the pore entrance, where pore–particle chemical interactions became relevant and often dominant.

We employ a continuum model for analyzing the full process, from bulk diffusion to pore docking.

### Generalized Smoluchowski Model

Let us consider a dilute solution of nanoparticles or biomolecules. The conservation equation for the solute concentration *C*(**r**,*t*) is
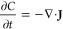
1where **J** is the flux that has three different components, (i) the diffusive, (ii) the phoretic, due to external forces acting on the solute particle, and (iii) the advection due to the solvent motion. In formulas
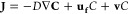
2with *D* denoting the diffusion coefficient, **u**_**f**_ the solvent flux velocity, and **v** velocity of the particle with respect to the fluid generated by the external forcing (for instance, the electrophoretic velocity). In particular, the latter will be expressed as **v** = μ**F**, with μ being the particle mobility and **F** the external force acting on the particle. Once suitable models are formulated for **v** (or **F**) and **u**_**f**_ and proper initial and boundary conditions are selected, eqs 1 and 2 constitute a system of partial differential equations that can be solved to get the time evolution of the concentration *C* and the flux **J** in the entire domain.

Here, we introduce some simplifications, allowing the analytical solutions of eqs 1 and 2 to be found for the particle capture in nanopores. We assume that the problem has a radial symmetry with respect to the center of the pore entrance (origin). Indicated by *r*, the distance from this origin, our domain is the semi-infinite hemisphere shell between *r* = *r*_e_ (entrance radius) and *r* → *∞* (see Figure 2b). Thanks to the radial symmetry, all the quantities involved in eqs 1 and 2 depend only on *r*. We also assume that the solvent flow satisfies the condition for incompressibility, ∇·**u**_**f**_ = 0. Furthermore, we suppose that the flux contributions associated with **u**_**f**_ and **v**, in eq 2, can be derived by differentiating a function ϕ(*r*) that is a dimensionless effective potential, *i.e.*, *∂ϕ*/*∂r* = (μ*F* + *u*_f_)/*D*, with *u*_f_ and *F* being the radial components of **u**_**f**_ and **F**. The explicit form of ϕ(*r*) and its connection to the models for electrophoresis and dielectrophoresis will be provided in the following when eqs 11–19 are discussed. Finally, we consider only cases where the diffusion coefficient *D* is homogeneous and constant. Consequently, eqs 1 and 2 reduce to
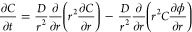
3that in the stationary case and integrating over *r* becomes
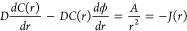
4with *A* being a constant to be determined from the boundary conditions. The integration of *J*(*r*) over a hemisphere of radius *r* leads to the particle capture frequency, *f* = 2π*A*.

The bulk concentration far from the pore (*r* → *∞*) is fixed at *C*_0_. We impose that the pore boundary, *r* = *r*_e_, is partially adsorbing (also referred to as radiative or Robin boundary condition) prescribing that only a fraction of the particles arriving at the boundary actually enters the pore (*i.e.*, successfully docks to the pore in Figure 2a), whereas the others are reflected. The process is controlled by the rate *k* > 0:
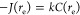
5The limit *k* → *∞* recovers the perfectly adsorbing condition *C*(*r*_e_) = 0.

The generic solution of the stationary problem (4) satisfying the boundary condition is
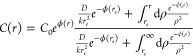
6From *C*(*r*), the flux density *J*(*r*) can be easily derived *via*eq 1, and it becomes *J*(*r*) = −*A*/*r*^2^.

The resulting capture frequency, that is, the number of particles per unit of time that are adsorbed at *r* = *r*_e_, is
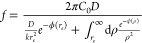
7Algebraic details are reported in Supporting Information S1.

Equation 7 nicely shows that the capture frequency can be split in two contributions, *f*_a_ due to the particles approaching the pore and *f*_e_ due to the particles actually entering the pore region:
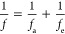
8with
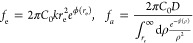
9In other words, defined the average capture time as τ = 1/*f*, we have
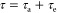
10with τ_a_ = 1/*f*_a_ being the average approaching time and τ_e_ = 1/*f*_e_ being the average entrance time. In the limit *k* → *∞*, corresponding to a completely adsorbing boundary, our results converge to the expression provided in ref (38). Moreover, if no external force is present, ϕ(*r*) = 0, the standard Smoluchowski expression, *f* = 2π*C*_0_*Dr*_e_, is recovered. In the following, we will specify the shape of the dimensionless effective potential ϕ used in this work.

### Model of Advection, Electrophoresis, and Dielectrophoresis

Let *Q*_*f*_ be the volumetric flow rate entering the pore, here assumed to be constant in time, the incompressibility of the solvent implies that
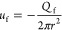
11In essence, eq 11 states that the integral of the velocity **u**_**f**_ over any hemisphere of radius *r* is equal to *Q*_f_. In actual systems, *Q*_f_ can be either induced by a pressure difference Δ*P* established between the two chambers connected by the pore, or it can be generated by electroosmosis.^28−30,50^ In the latter case, as a first approximation, we will use the Ohmic relation
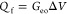
12where Δ*V* is the applied voltage across the electrolytic cell and the constant *G*_eo_, indicated in the following as electroosmotic conductance, may be estimated using the continuum electrohydrodynamics^51−53^ or computed by molecular dynamics simulations.^50,54^

The velocity **v** of the particle with respect to the fluid due to external forcing is expressed as **v** = μ**F**, with μ being the particle mobility and **F** being the external force induced by the electrical field. Electrophoretic and dielectrophoretic contributions read
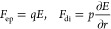
13where *q* and *p* indicate the particle charge and the intensity of its dipole, respectively. In *F*_di_, as a first approximation, we assumed that the dipole **p** is constant and oriented with the electrical field **E**. This amounts to considering the rotational diffusion negligible. Moreover, we are assuming that the permanent (intrinsic) dipole of the molecule is much larger than the dipole induced by the applied electric field **E**. This hypothesis is, somehow, reasonable for small molecules (*e.g.*, proteins and small peptides) for which the standard dielectrophoresis theory of colloids dramatically fails.^55,56^ If the permanent dipole is negligible, *F*_di_ scales, as usual, with ∇*E*^2^. This case is not discussed in this work, although the general result provided in eqs 6–10 can still be applied after a suitable model for the dimensionless potential ϕ(*r*) is provided.

The electric field **E** is derived using the hemispherical model for the pore entrance,^34,57^ according to which the electrical field intensity can be written as
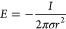
14where σ is the electrolyte conductivity and *I* is the ion current flowing through the pore. It is worth noting that, in a first approximation, *I* is proportional to σ; hence, once the pore geometry is specified and a suitable model for the pore conductance is selected, σ simplifies. The minus sign in eq 14 stems from the (arbitrary) choice of the ground electrode. Here, without loss of generality, we assume that the voltage is applied to the chamber where molecules are initially placed, whereas the other chamber is grounded. Thus, a positive voltage Δ*V*, corresponding to a positive current *I*, is associated with an electrical field directed toward the pore; as a consequence, the radial component of **E** is negative. Substituting eq 14 into expression 13, we get
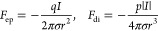
15Notice that the orientation of *F*_ep_ depends on the sign of *q* and *I*, whereas *F*_di_ always points toward the pore. This is a direct consequence of the assumption that the particle always orients its dipole **p** concordant with **E**, so it always moves in the direction of the increasing *E* and thus toward the pore.

The sum of the radial forces (15) and the advection contribution (11) can be expressed as the derivative of an effective potential:
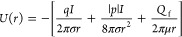
16where the latter term corresponds to the advection flow. Moreover, the notation is conveniently simplified by defining a dimensionless effective potential:
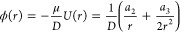
17with
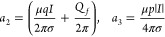
18The differentiation of ϕ(*r*) with respect to *r* yields the advection and electrophoretic contribution decaying as *r*^–2^ with a factor *a*_2_ and dielectrophoretic contribution decaying as *r*^–3^ with a factor *a*_3_.

### Approach Frequency ***f*_a_**

Interestingly, despite the combined effect of electroosmosis, electrophoresis, and dielectrophoresis, the approach frequency *f*_a_ assumes a simple analytical expression:
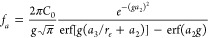
19with *g* = (2*Da*_3_)^−1/2^ and erf(*x*) being the error function. Formula 19 can be specialized in simpler expressions if either *a*_2_ or *a*_3_ (or both) are set to zero. In particular, when dielectrophoresis is absent (*a*_3_ = 0), because, for instance, the particle has a negligible dipole moment, we get
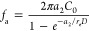
20whereas, when *a*_2_ = 0, because *Q*_f_ = 0 and dielectrophoresis is the only active forcing, like in the case of a neutral particle with a dipole moment, the capture frequency reads
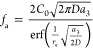
21Finally, if there is no external force and no fluid flow, *a*_2_ = *a*_3_ = 0, the only mechanism able to bring a particle near the pore entrance is the diffusion. In this case, the capture frequency is given by the standard Smoluchowski formula, *f*_a_ = 2π*DC*_0_*r*_e_. The derivation of eqs 19–21 can be found in the Supporting Information S1.

**Figure 3:**
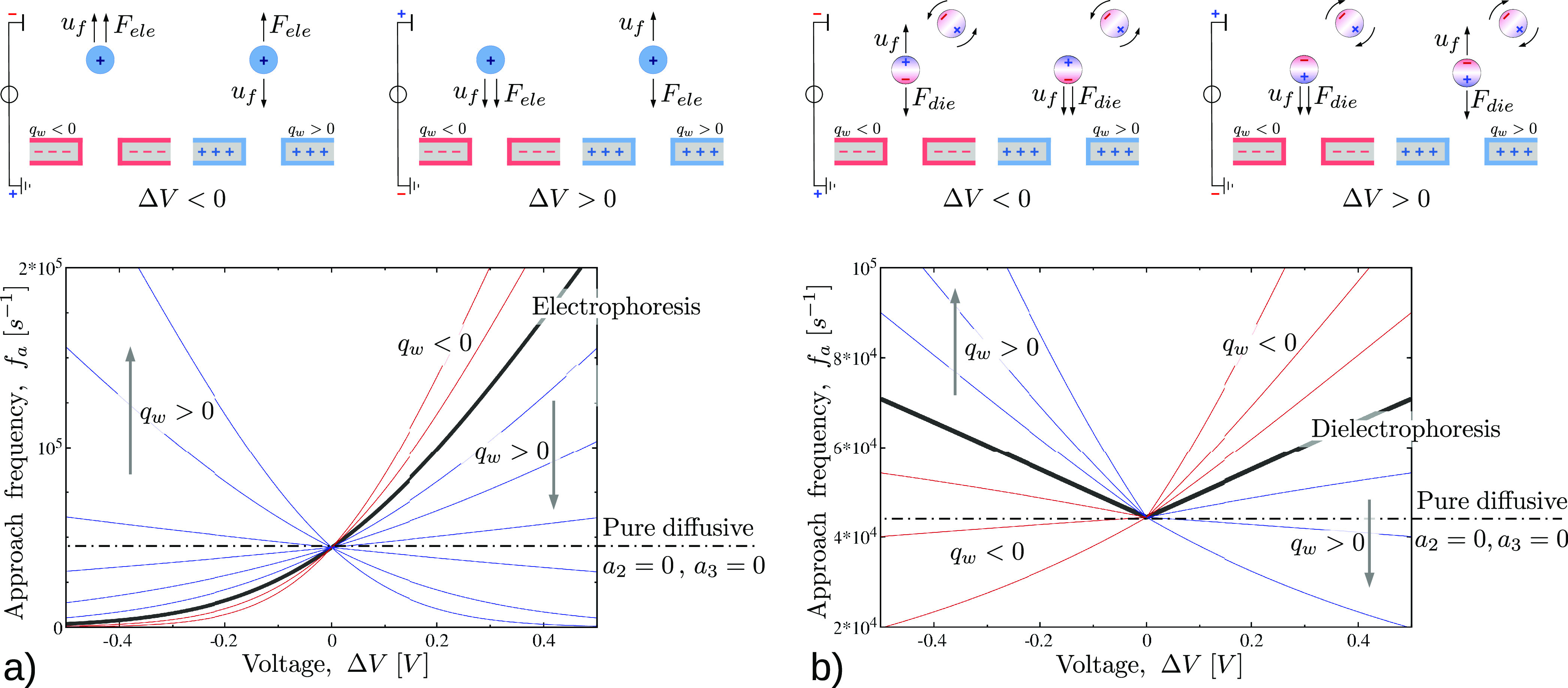
(a) Electrophoresis *vs* electroosmosis. Approach frequency, *f*_a_, in the absence of dielectrophoresis, *a*_3_ = 0, eq 20, as a function of the applied voltage Δ*V*. Particles (*C*_0_ = 40 μM) with a radius of 1.5 nm and charge *q* = 4*e* are immersed in a 1 M KCl electrolyte solution and captured into a pore of radius *r*_p_ = 2 nm and length *L* = 10 nm. Black line refers to the electrophoretic case (null advection, *Q*_f_ = 0). Blue lines correspond to positive surface charge, *q*_w_ = 0.012, 0.025, 0.037, 0.05, 0.075, and 0.1 C m^–2^. The connection between *q*_w_ and the electroosmotic current *Q*_f_ is given by eq 22. Red curves, instead, correspond to negatively charged pores *q*_w_ = −0.012, −0.025 C m^–2^. (b) Dielectrophoresis *vs* electroosmosis. Approach frequency, *f*_a_, in the presence of dielectrophoresis. Solid line refers to purely dielectrophoresis capture, *i.e.*, *a*_2_ = 0, eq 21. Pore shape, electrolyte solution, particles size, and concentration are the same as in panel (a). Now the particle is neutral *q* = 0, and it carries an intrinsic dipole *p* = 12 *e*·nm, *i.e.*, *p* ≃ 600 D. When the pore surface is charged, an electroosmotic flow sets in and the approach frequency *f*_a_ is given by eq 19. Blue and red lines correspond to positive and negative surface charge *q*_w_, namely, *q*_w_ = −0.025, −0.012, −0.006, 0.006, 0.012, 0.025 C m^–2^. For both panels, the horizontal black dotted line refers to the purely diffusive, *a*_2_ = *a*_3_ = 0, *i.e.*, *f*_a_ = 2π*r*_e_*C*_0_*D*.

The first examples of the approaching frequency, *f*_a_, as a function of the applied voltage Δ*V* are represented in Figure 3a for a cylindrical pore with a radius *r*_p_ = 2 nm and length *L* = 10 nm. The electrolyte is a 1 M KCl solution with conducibility σ = 11.1 S/m and Debye length λ_D_ = 0.3 nm. The particle concentration is *C*_0_ = 40 μM. The pore resistance, *R*_tot_, can be calculated as a combination of the pore and access resistance, *R*_tot_ = *R*_p_ + 2*R*_a_, where *R*_p_ = *L*/(π*r*_p_^2^σ) is estimated by a simple quasi-1D model and *R*_a_ = 2(σ*r*_p_)^−1^ is estimated in the hemispherical electrode approximation.^34,57^ Hence, the total resistance becomes *R*_tot_ ≃ 0.12 GΩ. We considered the entrance radius *r*_e_ coinciding with the pore radius *r*_p_.

The particles to be absorbed are spheres of radius of *a* = 1.5 nm, so their mobility is estimated by the Stokes law as μ = (6*πaνρ*)^−1^, with ρ the density of water, and ν = 10^–6^*m*^2^/*s* the kinematic viscosity of water. The diffusion coefficient is hence calculated using the fluctuation–dissipation relation *D* = *k*_B_*Tμ*, with *k*_B_ the Boltzmann constant and *T* the temperature. At first, we considered positive particles of charge *q* = 4*e* under the combined effect of electrophoresis and electroosmosis, so the approach frequency *f*_a_ is given by eq 20. The solid line in Figure 3a refers to the pure electrophoretic case. As expected, *f*_a_ increases with Δ*V* > 0, whereas it strongly reduces for Δ*V* < 0. For a comparison, the purely diffusive frequency, *f*_a_ = 2π*r*_p_*DC*_0_, is also reported as an horizontal dotted-dashed line.

We then considered the case where the pore carries a surface charge *q*_w_ which, under several simplifying hypothesis (circular cylinder, no entrance effect, no-slip wall, *r*_p_ ≫ λ_D_; see the Supporting Information S2 for details), provides the conductance
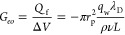
22The approach frequencies, *f*_a_, for positive and negative surface charge, *q*_w_, are plotted as blue and red lines in Figure 3a. For a negatively charged surface, *q*_w_ < 0, and positively charged particles, electroosmosis cooperates with electrophoresis. Indeed, a negative surface charge is able to induce an accumulation of positive ions in the pore, and hence, the electroosmotic flux is directed as the electric field. Therefore, for Δ*V* > 0, the electroosmotic flux increases *f*_a_, whereas, for Δ*V* < 0, it reduces *f*_a_ with respect to the pure electrophoretic case. Just the opposite occurs for *q*_w_ > 0; now electroosmosis competes with electrophoresis and positive values of Δ*V* reduce *f*_a_. For large *q*_w_, the electroosmotic flux is so intense to overwhelm the electrophoresis. As a consequence, capture is favored at negative voltages. This phenomenon was recently observed by Asandei *et al.*(30) in the capture of peptides by biological pores, where a large positive surface charge is induced by decreasing the pH of the solution. Similar evidence was reported in ref (58), where a globular protein is captured by the ClyA pore against electrophoresis.

**Figure 4:**
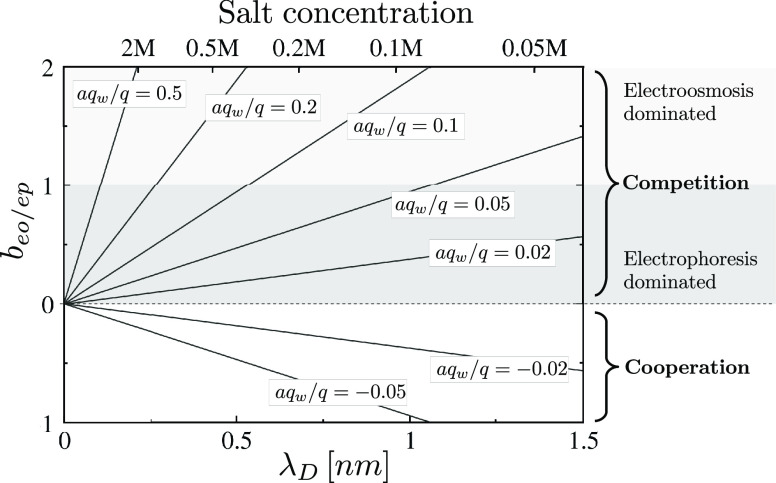
Competition/cooperation between electroosmosis and electrophoresis. The dimensionless parameter *b*_eo/ep_, eq 23, representing the ratio of electroosmotic over electrophoretic transport contribution, is reported as a function of the Debye length λ_D_ for different values of *aq*_w_/*q*, with *q*_w_ being the wall surface charge density, *q* the particle charge, and *a* its radius. The upper horizontal axis represents the concentration of a unitary valence salt (*e.g.*, KCl or NaCl) corresponding to λ_D_.

From both electrophoretic and electroosmotic terms appearing in the expression of *a*_2_, eq 18, we can define the dimensionless parameter:
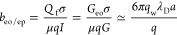
23where, in the last equality, we used eq 22 to compute *G*_eo_, the Stokes expression for the mobility of a spherical particle and assumed that the pore ionic conductance *G* = 1/*R* does not include the access resistance. Figure 4 reports *b*_eo/ep_ as a function of the Debye length, λ_D_. For *b*_eo/ep_ < 0, electroosmosis and electrophoresis cooperate, whereas when *b*_eo/ep_ > 0, they compete; that is, they act in opposite direction on the molecules. This competition can be dominated either by electrophoresis, 0 < *b*_eo/ep_ < 1, or by electroosmosis, *b*_eo/ep_ > 1. In the example of Figure 3a, the maximum positive surface charge *q*_w_ = 0.1 C m^–2^ corresponds to *b*_eo/ep_ ≃ 1.4 (*i.e.*, electroosmosis larger than electrophoresis), the smaller positive one *q*_w_ = 0.012 C m^–2^ corresponds to *b*_eo/ep_ ≃ 0.16 (*i.e.*, electrophoresis drives the process), whereas for pore surface charge *q*_w_ < 0, electroosmosis and electrophoresis cooperate. Note that, basically, the parameter *b*_eo/ep_ is the ratio between the amplitude of the electroosmotic velocity, **u**_**f**_, and the electrophoretic velocity, **v**, induced by the external forcing.

A second example concerns the dielectrophoretic capture and its competition with electroosmosis (see Figure 3b). Now the particle has no net charge (*q* = 0), whereas it carries a dipole *p* = 12*e*·nm, *i.e.*, *p* ≃ 600 D; this value is in the typical dipole range for proteins.^59^ If no advective flow is present, the approach frequency *f*_a_ is given by eq 21 (black solid curve). At both positive and negative Δ*V*, the frequency *f*_a_ is larger than the diffusive case. Moreover, the curve *f*(Δ*V*) is symmetric, thus dielectrophoresis favors the capture in the same way at both positive and negative voltages. If the pore surface is charged, an electroosmotic flow sets in. Following the same approximations used for Figure 3a, the electroosmotic conducibility is expressed as a function of the pore surface charge *q*_w_*via*eq 22. Blue and red curves of Figure 3b refer to *q*_w_ > 0 and *q*_w_ < 0, respectively. Comparing the electroosmotic and dielectrophoretic term in ϕ(*r*), eq 17, we can define the dimensionless parameter:
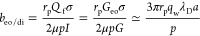
24where, again, we used expression 22 for *G*_eo_. For the smaller *q*_w_ in Figure 3b, we get *b*_eo/di_ ≃ 0.02; that is, the electroosmosis does not qualitatively affect the particle kinetics, and *f*_a_ is larger than the pure diffusive case at both Δ*V* > 0 and Δ*V* < 0. As *q*_w_ increases, electroosmosis starts influencing the process, and for *q*_w_ = 0.025 C m^–2^, *b*_eo/di_ ≃ 0.1. It is worth noting that *b*_eo/di_ expresses the ratio of electroosmotic and dielectrophoretic effects at the pore entrance (*r* = *r*_e_). However, as electroosmosis scales as *r*^–2^ and dielectrophoresis as *r*^–3^, far enough from the pore, electroosmosis soon or later prevails, even though *b*_eo/di_ is small.

### Entrance Frequency *f*_e_

Let us consider here the entrance frequency *f*_e_ defined in eq 9 and the corresponding average entrance time τ_e_ = 1/*f*_e_, repeated for convenience:
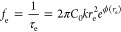
25A few preliminary remarks are in order. First, *f*_e_ is affected by the value of the dimensionless effective potential ϕ evaluated at the pore entrance, *r* = *r*_e_. If ϕ(*r*_e_) is positive, *e.g.*, both *a*_2_ > 0 and *a*_3_ > 0, then *e*^ϕ(*r*_e_)^ > 1. This has a direct physical interpretation; indeed, *a*_2_ > 0 and *a*_3_ > 0 correspond to particle velocities directed toward the pore and, hence, to an increase of the entrance frequency. The opposite occurs when ϕ(*r*_e_) < 0 (*e*^ϕ(*r*_e_)^ < 1); the particle velocity is directed away from the pore and, hence *f*_e_ decreases.

Second, for *k* → *∞* (perfectly adsorbing boundary), we get *f*_e_ → *∞* and τ_e_ → 0. This reflects the occurrence that for an adsorbing boundary, any time a particle hits the pore entrance, it is instantaneously captured (average entrance time τ_e_ = 0). The capture frequency *f*, eq 8, and the average capture time *f* = 1/τ, eq 10, therefore reduce to the approach values, *f*_a_ and τ_a_, whose expressions were derived in the previous section. We will refer to this case as the transport-limited regime, as *f* is governed only by diffusion, advection, and external forces (see Figure 1a) and not by the atomistic details of the pore docking.

In the opposite condition, *k* → 0, *f*_e_ → 0, and τ_e_ → *∞*, consequently, the capture frequency *f* vanishes. Again, this is expected as for *k* → 0, we are considering the limiting condition of a perfectly reflective boundary. The interesting regime occurs for finite *k*, and for analyzing this regime, we first need to formulate a reasonable model for *k*.

### Model for the Absorption Rate, *k*

When a particle engages the pore entrance, chemical details related to particle and pore compositions start to play a crucial role. We dubbed this stage as pore docking in Figure 2, and its accurate modeling necessarily requires an atomistic description of the system. Nevertheless, here, we attempted to model the pore docking and, in particular, the constant *k* appearing in the Robin boundary condition, eq 5.

We assumed an Arrhenius-like form:
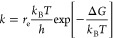
26where an Eyring-like expression for the prefactor is used with *k*_B_ = 1.38 × 10^–23^ J K^–1^ being the Boltzmann constant, *h* = 6.62 × 10^–34^ Js the Planck constant, and *T* the absolute temperature. Δ*G* represents the free-energy barrier, generally positive, to be overcome by the particle in order to enter the pore. High values of Δ*G* reduce exponentially the adsorption probability.

If the particle is pushed toward the pore by advection or other external fields, it can cross the barrier more easily. We modeled this effect by writing Δ*G* as
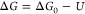
27where Δ*G*_0_ is the free-energy barrier at equilibrium and *U* is the barrier reduction (or increase) due to electric and advection effects in the docking phase. Accordingly, eq 25 reads
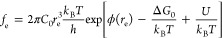
28

### Transport-Limited and Entrance-Limited Regimes

**Figure 5:**
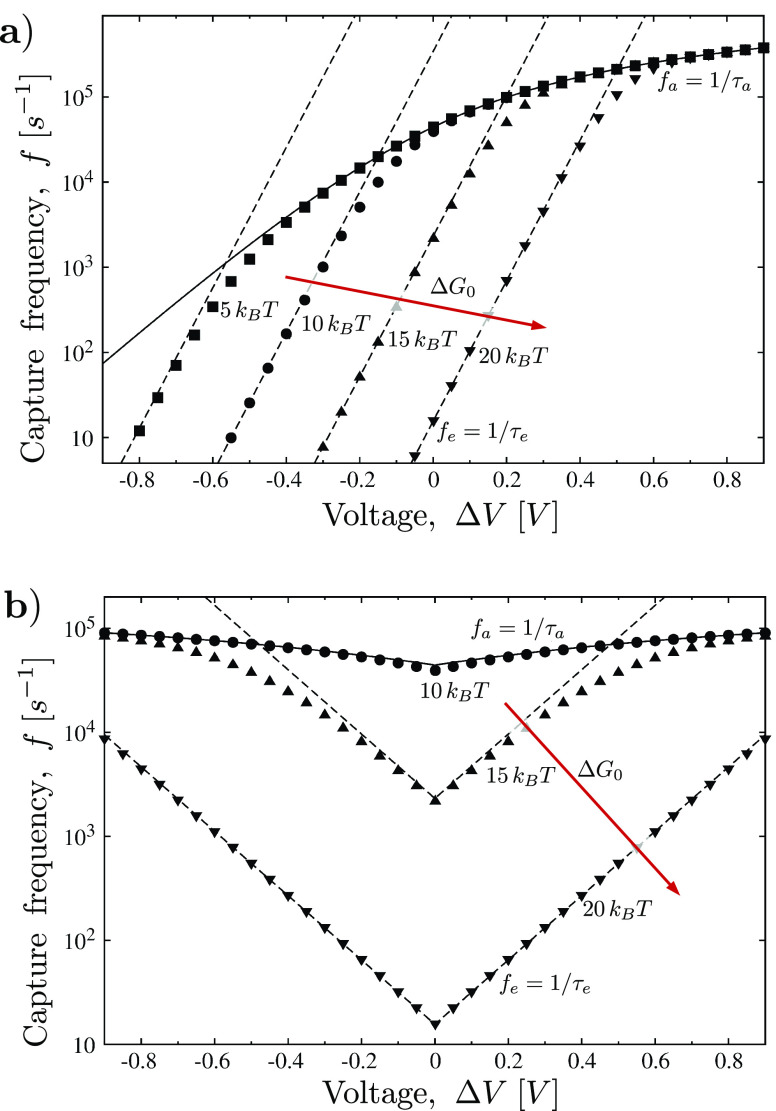
Effect of entrance barrier on capture frequency. (a) Electrophoretic case, *a*_3_ = 0. Capture frequency *f* as a function of Δ*V*. The pore and the particle are the same as in Figure 3a in the absence of electroosmosis. The entrance model is given by eq 29. The equilibrium barrier is Δ*G*_0_ = 5, 10, 15, and 20 *k*_B_*T* for squares, circles, up-triangle, and down-triangles, respectively. Dashed lines refer to entrance frequency *f*_e_ for the four Δ*G*, whereas the solid line refers to the approach frequency *f*_a_. (b) Dielectrophoretic case, *a*_2_ = 0. The pore and the particle are the same as in Figure 3b in the absence of electroosmosis. The same line and point style of previous panel is used. Δ*G*_0_ = 5 *k*_B_*T* is not reported as it overlaps with Δ*G*_0_ = 10 *k*_B_*T*. Examples of the corresponding concentration profiles are reported in Figure S1 in the Supporting Information.

As examples, we discuss here the same system considered in Figure 3a,b, where now we include also the entrance effects. We assume *U* = *k*_B_*Tϕ*(*r*_e_) so that the resulting entrance frequency, eq 28, becomes
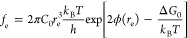
29Figure 5a displays the electrophoretic case (*a*_3_ = 0 and *G*_eo_ = 0). For large Δ*V*, the entrance barrier does not affect the capture, and *f* ≃ *f*_a_. Indeed, in this case, ϕ(*r*_e_) = *a*_2_/(*Dr*_e_), so that ϕ(*r*_e_) linearly increases with Δ*V*. Accordingly, for large Δ*V*, *f*_e_ → *∞* and τ_e_ = 1/*f*_e_ → 0 that, when used in eqs 8 and 10, gives *f* = *f*_a_. A reduction of Δ*V* increases τ_e_ until the process is dominated by the entrance barrier. A similar analysis is performed for the dielectrophoretic case (Figure 5b). Now, as already discussed, the capture frequency plot is symmetric, *i.e.*, *f* = *f*(|Δ*V*|). For large |Δ*V*|, we have *f* ≃ *f*_a_, whereas for small |Δ*V*|, the capture is controlled by the height of the entrance barrier and *f* ≃ *f*_e_.

Hence, in both cases, by changing Δ*V*, we pass from a regime where the barrier does not have any effect on the capture (transport limited regime, τ_a_ ≫ τ_e_) to a condition where the capture is dominated by the energy barrier at the entrance (entrance limited regime, τ_a_ ≪ τ_e_). The threshold between the two regimes depends on the value of the equilibrium barrier Δ*G*_0_ and on the forces acting on the particle. An interesting outcome of our work is that, as we have explicit analytical expressions for τ_a_ and τ_e_, we have a quantitative tool to assess if the process is transport-limited or entrance-limited.

### Comparison with Experiments

Our model provides a guideline to interpret observed capture frequency in nanopore experiments. Here, we first illustrate some comparisons with the literature, and then we discuss a dedicated dielectrophoretic capture experiment we performed.

### Comparison with Literature

In previous sections, we analyzed the competition between electrophoretic and electroosmosis (Figure 3a) and its connection with the work.^30^ In ref (30), the capture of a positively charged peptide was observed against electrophoresis into a biological pore (α-hemolysin, αHL) whose anion selectivity was increased by altering the pore surface charge through a reduction of the solution pH to pH 2.8. Other evidence of electroosmotic capture is reported in Huang *et al.*,^29,60^ where proteins and peptides of both positive and negative charge are captured by the FraC biopore. In these examples, the size of the pore entrance is comparable with the molecule, and both the pore and the molecule have a complex charge distribution. Hence, we expect that atomistic details strongly affect the capture frequency *f*.

We analyze here in details the experiment by Asandei *et al.*(30) A first question concerns whether the process is in the transport-limited or entrance-limited regime. This question can be answered by calculating the average approach time τ_a_ = 1/*f*_a_ and comparing it with the experimental average capture time τ. The simplest estimation for τ_a_ is given by the purely diffusive expression *t*_a_ = 1/*f*_a_ = (2π*C*_0_*Dr*_e_)^−1^. We set *C*_0_ = 30 μM (as in ref (30)) and *r*_e_ = 1 nm (from the αHL crystal structure^45^). The diffusion coefficient is calculated as *D* = μ*k*_B_*T*, where the mobility is given by μ = (6π*N*νρ*a*)^−1^, with *N* = 20 being the number of amino acids forming the peptide, ν and ρ are the water kinematic viscosity and density, and *a* = 0.4 nm is the typical hydrodynamic radius of a single amino acid. The resulting value is τ_a_ ≃ 3 × 10^–4^ s. As the average capture time from Figure 4a in Asandei *et al.*(30) is τ ∼ 1 s, in light of our model, we deduced that τ_e_ ≫ τ_a_, that is, the main bottleneck in the capture is represented by the entrance barrier. The capture is in the entrance-limited regime, where peptides easily reach the pore entrance but hardly overcome the barrier. This implies that, to get a quantitative *a priori* prediction on the capture rate, we need a detailed model of the pore-docking process. In the absence of that, yet our model can provide a useful guideline to assess which mechanism among electrophoresis and electroosmotic is dominant (the peptide in ref (30) has a negligible dipole, so dielectrophoresis is excluded). In particular, we can calculate the ratio *b*_eo/ep_ from eq 23. Concerning pore and electrolyte properties, we used *G*_eo_ ≃ 1.6 m^3^/V, from molecular dynamics simulations at pH 2.8 in ref (50) and *G* ≃ 2.2 from ref (30) and σ = 20 S/m. Concerning the peptide properties, the total charge *q* = 8*e* is used. Putting together all of these ingredients, we get *b*_eo/ep_ = 1.3, *i.e.*, electroosmosis dominates the process, coherently with the experimental findings.

**Figure 6:**
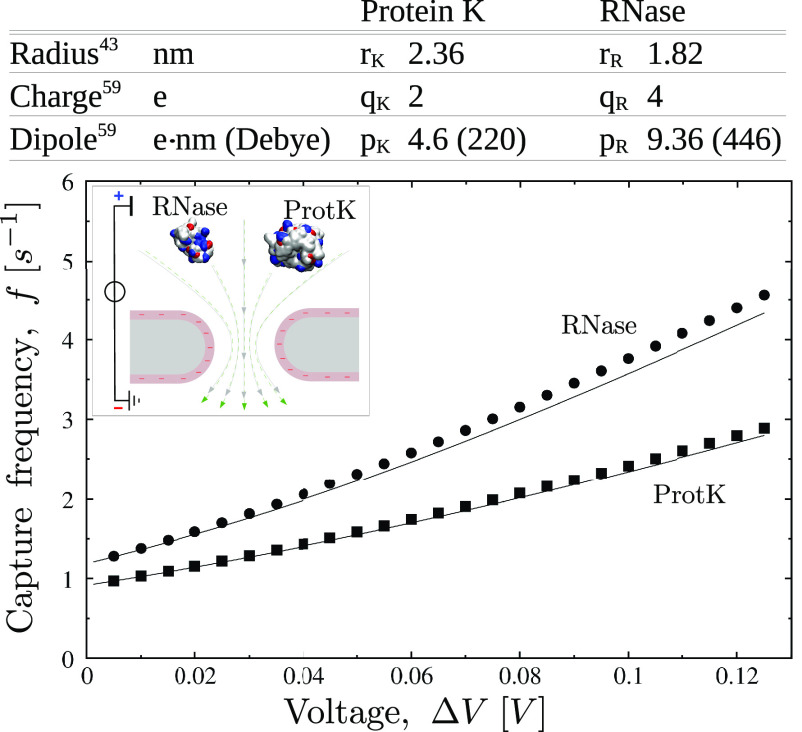
**Capture frequency for Larkin et al. experiment.**(43) Circles and squares refer to theoretical capture frequency calculated via eq 19 for RNase A and Protein K. Lines refer to eq 20 (dielectrophoresis neglected). In both cases, protein bulk concentration is *C*_0_ = 1 nM and complete adsorption is assumed (τ_e_ = 0 and *f* = *f*_a_, transport limited regime). The pore is slightly negatively charged at the experimental pH = 8.1 while both proteins are positive, so, electroosmosis and electrophoresis cooperate at Δ*V* > 0. Protein sketches are created using VMD,^49^ blue and red areas refer to positively and negatively charged residues.

To get a quantitative prediction of the capture frequency in biological pores, a reliable model of the pore-docking process is needed. This task is out of the aim of the present work as, inevitably, it will depend on the specific pore–particle interaction. Hence, for a quantitative assessment of our model, we selected the experiment by Larkin *et al.*,^43^ in which the pore entrance is slightly larger than the particle and the captured particles are not flexible. These conditions suggest the entrance effect should be negligible. The experimental setup of ref (43) is constituted by a solid-state hafnium dioxide (HfO_2_) pore of radius *r*_p_ ≃ 2.6 nm, length *L* = 7 nm, and conductance *G* ≃ 20 nS. The experiment is conducted in a 1 M KCl solution at pH 8.1, where the HfO_2_ pore is slightly negatively charged. We estimated an electroosmotic conductance *G*_eo_ ≃ 15 × 10^–18^ m^3^/s *via*eq 22, where for the surface charge we used *q*_w_ = −0.025 C/m^2^ estimated from the data of ref (61), and the Debye length is λ_D_ = 0.3 nm. The authors of ref (43) studied the capture of two globular proteins, ProK and RNase A, whose data are taken from previous studies^43,59^ and are summarized in Figure 6.

Figure 6 plots the capture frequency wherein we neglected the entrance contribution, *i.e.*, *f*_e_ = 0 and *f* = *f*_a_, for protein concentration *C*_0_ = 1 nM. Points refer to eq 19 that includes electrophoresis, electroosmosis, and dielectrophoresis, whereas lines indicate that dielectrophoresis is set to zero, *i.e.*, *a*_3_ → 0, eq 20. It is apparent that the dielectrophoretic contribution, in this case, is negligible. Moreover, as the pore is negatively charged, for positive Δ*V*, electroosmosis is directed toward the pore so that it cooperates with electrophoresis. Our capture frequencies are 4–5 times smaller than the ones observed in the experiments (see Figure 3 of ref (43)). As our model has no adjustable parameters, such a prediction can be considered good. Nevertheless, it can be useful to list some possible causes of the discrepancy: (i) the surface charge estimation *q*_w_ could not be accurate. The data we used^61^ refer to a 0.1 M NaCl solution, while the experiment was run at 1 M KCl. Even assuming that the salt composition does not affect *q*_w_, the concentration is quite different and Figure 2 of ref (61) suggests that larger salt concentrations correspond to greater *q*_w_. Hence, *q*_w_ is likely underestimated. (ii) When the molecule gets closer to the pore entrance, it induces a current reduction, which is associated with a local increase of the resistance and, accordingly, to an enhancement of the local electric field *E*. Easily, this enhancement can bring a factor 3 to the field strength, with respect to our estimation (see Supporting Information S3). (iii) The analysis of the protonation state of protein titrable residues, when calculated with tools that take into account the local environment,^62^ provided charge values slightly larger than those we used, in particular, *q*_K_ = 3*e* and *q*_R_ = 5*e*. All of those factors would increase the predicted capture frequency, and presumably, they qualitatively explain why our model underestimates it.

### Experimental Results for Dielectrophoretic Capture

**Figure 7:**
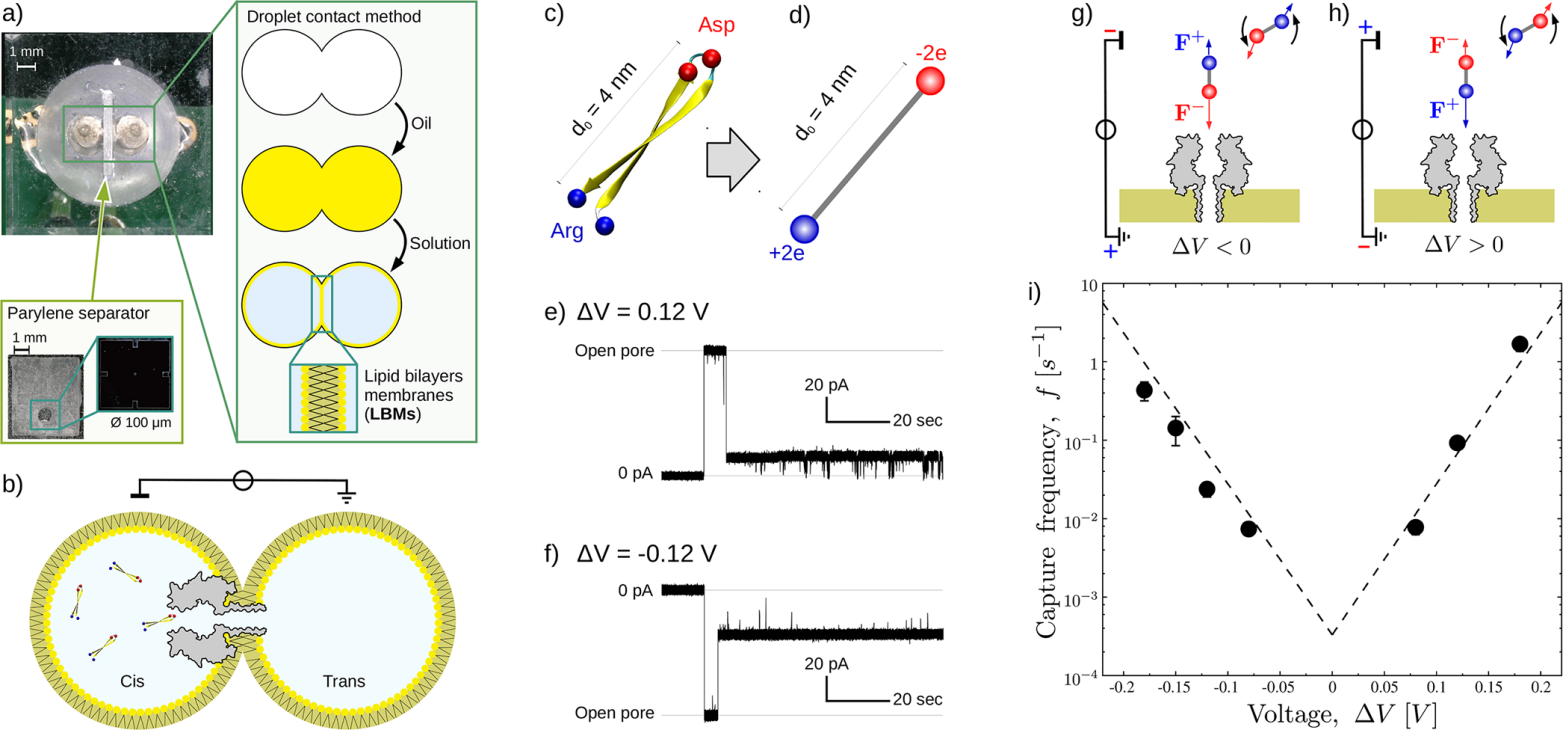
**Dielectrophoretic capture, experimental results.** (a) A two-droplet system is prepared using the droplet contact method.^63^ A single α-hemolysin, αHL, nanopore connects the two droplets. A sketch of the system (not in scale) is reported in panel (b). (c) The cis droplet contains the overall neutral β-hairpin peptides of 28 amino acids carrying two positive (Arginine, Arg) and two negative (Aspartic acid, Asp) charges at its two extremities. When a voltage Δ*V* is applied between the droplets, blockade events are observed for both positive (e) and negative (f) Δ*V*. In both cases, the proposed capture mechanism is the dielectrophoretic capture: the peptide is described as a rigid dipole dumbbell (d), the electrical field induces a torque that aligns the dipole along the electrical field. After the alignment, the unbalance in the forces acting on the positive (*F*^+^) and negative (*F*^–^) extremities, results in a net force attracting the molecule toward the pore, panels (g) and (h). Panel (i) reports the experimentally observed capture frequency *f*, black points. Line refers to theoretical estimation, eq 29 with Δ*G*_0_ = 22*k*_B_*T* and correction factor *s* = 10. Panel (c) was realized using the VMD software.^49^

For dielectrophoretic capture, we conducted dedicated experiments whose setup is sketched in Figure 7a,b. As a model for a rigid particle, we designed a peptide whose structure is a β-hairpin (see Figure 7c and Supporting Information S4 for structure prediction). At pH 7, the peptide is neutral. The turn of the peptide containing two aspartic acids (Asp) is negatively charged, whereas the terminals hosting two arginines (Arg) are positive. All of the other residues are neutral. The length of the peptide is *d* ≃ 4 nm. With reference to Figure 7d, our modeling portrays the peptide as a dumbbell of length *d*, with global charge *q* = 0, and dipole *p* = 8*e*·nm.

A few details follow on the system setup used for the capture frequency measurement. An αHL nanopore is embedded in a lipid bilayer membrane that is formed using the droplet contact method.^63^ The β-hairpin peptides are present only in one of the two droplets. A voltage applied between the two droplets, Figure 7b, induces a ionic current. When the β-hairpin enters the pore, the current intensity decreases, this event is indicated as current blockade. Details on the system preparation are reported in the method section. Current traces are represented for both positive and negative voltages in Figure 7e and Figure 7f. and blockade events are observed for both cases. This means that capture occurs independently of the voltage polarity, in qualitative agreement with the prediction of our dielectrophoretic capture model, Figure 3b. The proposed capture mechanism is sketched in Figures 7g and 7h. The capture time τ is calculated as the average of the duration of the open levels, see methods. The capture frequency *f* = 1/τ as a function of Δ*V* is shown in the Figure 7i and it nicely resembles the V-shaped curve typical of dielectrophoretic capture, Figure 3b and Figure 5b. As in the example of Asandei and co-workers^30^ previously discussed, the capture is dominated by the pore-docking phase and, hence, τ_a_ ≪ τ_e_ (entrance limited regime). Consequently, a reliable a priori quantitative prediction of *f* would need a proper modeling of the free-energy barrier Δ*G*_0_ and its alteration *U* due to the external forces appearing in eq 28. The formulation of an exact description is beyond the purpose of this work, nevertheless, we propose an ad hoc model able to reproduce the data. We applied eq 29 to calculate the entrance frequency *f*_e_ using *r*_e_ = 1 nm for pore entrance and *G* = 2 nS for pore conductance. The ad hoc effect we added to eq 29 is the increase of the local electric field, due to the current reduction produced by the peptide when engaging the pore. This amounts to multiplying *a*_3_ by a factor *s* > 1. Dashed line in Figure 7i refers to Δ*G*_0_ = 22 *k*_B_*T* and *s* = 10. A toy model for the estimation of the factor *s* is reported in Supporting Information S3.

## Conclusion

We have presented an analytical description of the capture process of nanoparticles in nanopores which embodies, in a unified framework, all the relevant stages: bulk diffusion, funneling and, finally, pore-docking. Our formulation is based on a Smoluchowski-like equation for the particle capture, where the approach to the pore is naturally described in terms of advection, diffusion and particle flux induced by external forces. In our formulation the entrance process is described as a partial adsorbing (Robin) boundary condition. Interestingly, the average capture time τ results to be the sum of an *approaching time*, τ_a_, due to the migration of particles from the bulk to the pore mouth, and, of an *entrance time*, τ_e_, associated with free-energy barriers the particles experience at the pore entrance. This result easily allows to quantify the relative weight of the approaching and the entrance stages in the capture process. Indeed, the ratio τ_a_/τ_e_ provides the natural dimensionless parameter to distinguish a *barrier limited regime* (τ_a_/τ_e_ ≪ 1) from a *transport limited regime* (τ_a_/τ_e_ ≫ 1).

We applied our general theory to analyze the interplay among three main electro-hydrodynamic effects involved in the particle capture at the nanoscales, namely, electroosmosis, electrophoresis and dielectrophoresis. Suitable models for these three effects allowed us to derive explicit analytical expression for the concentration profiles and the capture frequency. We first compared our theoretical predictions with some literature results on electrophoresis and electroosmotic capture. Finally, for dielectrophoresis, we conducted a dedicated experiment to confirm the main outcome of our model. In particular, using a single molecule nanopore-sensing system based on the lipid droplet method, we showed that a rigid dipole can be captured by a nanopore under both positive and negative voltages. The capture frequency increases with the amplitude of the applied voltage as predicted by our theoretical analysis.

When a continuum model is applied at nanoscale, questions arise on the underlying hypothesis and, consequently, on the reliability of the quantitative results. On the top of the standard continuum assumption, in our case, the strongest additional hypothesis is that the fields are radial outside the pore. In our formulation, this assumption is essential to get analytical results but numerical simulation showed that it is violated close to the pore, see, for instance, the electroosmotic and the electric field reported in.^52,53^ Since the region near the pore is crucial for both the funneling and the pore-docking phases, a fully 3D (or 2D axisymmetric) simulation including a more accurate estimation of the velocity and electric field could predict capture rates that may differ from our results. Another assumption used in our study is that the diffusion coefficient is homogeneous while confinement effects may be expected close to the pore. Including these effects, again, would necessarily call for numerical approaches based, for instance, on Brownian dynamics model with proper formulations to take into account the inhomogeneity of the diffusion coefficient.^64,65^

Our results can be of immediate application to the development of nanopore based technologies for blue-energy harvesting, water desalination and single molecule sensing. Concerning the latter point, our model can be useful in particular for protein sensing since the large variety of sizes, net charges and dipoles occourring in the proteome makes the interplay among electrophoresis, electroosmosis and dielectrophoresis not always trivial to disentangle. Moreover, the analytical solution we derived for the Smoluchowski equation with Robin boundary conditions, may impact other research fields too. Indeed, advection-diffusion equation in spherical coordinates under radial potential are commonly employed in a large variety of problems such as cellular nutrient uptake^66^ and aggregation limited processes.^67^

## Methods

Here, we report the details concerning the experimental setup used to get dielectrophoretic capture data described in Figure 7.

### Reagent and chemicals

Aqueous solutions were prepared with ultrapure water from a Milli-Q system (Millipore, Billerica, MA, USA). The reagents were as follows: 1,2-diphytanoyl-*sn*-glycero-3-phosphocholine (DPhPC; Avanti Polar Lipids, Alabaster, AL, USA), *n*-decane (Wako Pure Chemical Industries, Ltd., Osaka, Japan), potassium chloride (KCl; Nacalai Tesque). An α-hemolysin (αHL, Sigma–Aldrich, St. Louis, MO, USA) obtained as a monomer protein isolated from *Staphylococcus aureus* in the form of a powder was rehydrated at a concentration of 1 mg/mL in ultrapure water and stored at −80 °C. The β-hairpin peptide, SV28, sequence RGSYSVSVSVSYDSDGSYSVSVSVSYGR, was chemically synthesized as recently reported.^68^ As measurement solutions, a cis solution (1 M KCl, 10 mM MOPS, 100 nM αHL, 50 nM SV28, pH = 7.0) and a trans solution (1 M KCl, 10 mM MOPS, pH = 7.0) were prepared.

### Channel current measurement and data analysis

DPhPC (10 mg/mL) were dissolved in *n*-decane (0.5 μL) and the measurement solutions (each 4.7 μL) were added to both wells of the microfabricated device, in Figure 7a. The lipid bilayer membrane is formed with the droplet contact method.^63^ Channel current was measured using a Pico patch-clamp amplifier (Tecella, Foothill Rantch, CA) under an applied voltage of Δ*V* = 80, 120, 150, and 180 mV. Other measurement conditions were as follows: a sampling rate of 40 kHz, a Bessel filter of 15 kHz, and a gain of 10. All measurements were conducted at room temperature. The current value was recorded with Tecella Lab v 0.98 (Tecella, Foothill Rantch, CA). The analysis of channel current signals and the duration time of the current blockades were performed using pCLAMP ver. 10.5 (Molecular Devices, CA, USA) and Python.^69^ The current signals were converted to blocking ratio *b* = (*I*_0_ – *I*_b_)/*I*_0_, where *I*_0_ and *I*_b_ are the open and the blockade current levels, respectively. A blocking event was defined when blocking ratio *b* > 0.5 for a duration time >1 s. Average capture time τ was defined as the average of the open level duration distribution. Statistical error on τ was calculated as , where σ is the standard deviation of the capture time distribution and *N* is the number of events. Capture frequency is calculated as *f* = 1/τ, with error bars in Figure 7i being obtained from ϵ using standard propagation of uncertainty.

## Supporting Information Available

Stationary solution of advection–diffusion equation for radiation boundary condition, estimation of the electroosmotic flow for a cylindrical pore, expression of the local increase of electric field at the pore entrance due to blockade, and SV28 peptide structure prediction (PDF)
