# Supplementary material for: Analytical Model for Particle Capture in Nanopores Elucidates Competition among Electrophoresis, Electroosmosis, and Dielectrophoresis
Source: ACS Nano. 2020 Nov 10;14(11):15816–28. doi: 10.1021/acsnano.0c06981 (PMC8016366; doi:10.1021/acsnano.0c06981)
Supplement: Supplementary file 1 — nn0c06981_si_001.pdf [file nn0c06981_si_001.pdf]

Supplementary Information for: Analytical Model for Particle Capture in Nanopores Elucidates Competition among Electrophoresis, Electroosmosis and Dielectrophoresis.

Mauro Chinappi,[*,†] Misa Yamaji,[‡] Ryuji Kawano,[‡] and Fabio Cecconi[*,¶]

†*Dipartimento di Ingegneria Industriale, Università di Roma Tor Vergata, Via del Politecnico 1, 00133 Roma, Italia.*

‡*Department of Biotechnology and Life Science, Tokyo University of Agriculture and Technology, Tokyo 184-8588, Japan*

¶*CNR-Istituto dei Sistemi Complessi, Via dei Taurini 19, I-00185 Rome, Italy.*

E-mail: mauro.chinappi@uniroma2.it; fabio.cecconi@roma1.infn.it

Supplementary note S1: Stationary solution of advection-diffusion equation for radiation boundary condition

In this section of SI, we present in details the solution of the stationary Eq. (4) of the paper, that we report for convenience,

$$D\frac{dC(r)}{dr} - DC(r)\frac{d\phi}{dr} = \frac{A}{r^2} \tag{S1}$$

leading to the derivation of formula (7) of the main tex for the capture frequency. The domain of this solution is the semi-spherical shell between $r = r_e$ (entrance radius) and $r = r_b$ (bulk radius), with boundary conditions, $C(r_b) = C_0$, C_0 being the particle bulk concentration and the radiation (or Robin) boundary condition (RBC) near the pore entrance,

$$-J(r_e) = kC(r_e) \; . \tag{S2}$$

The limit $k \to \infty$ recovers the perfectly adsorbing condition at the pore entrance, $r = r_e$, while $k \to 0$ corresponds to a no-flux boundary. Using Eq. (S1), the RBC can be recast to

$$D \frac{\partial C(r)}{\partial r}\bigg|_{r=r_e} - DC(r_e)\frac{d\phi(r)}{dr}\bigg|_{r=r_e} = kC(r_e) \; . \tag{S3}$$

Since Eq. (S1) is linear, its general solution can be found by combining the solution of the homogeneous equation ($A = 0$) and a particular solution of the complete equation ($A \neq 0$). The homogeneous equation is easily solved,

$$C_h(r) = B \exp[\phi(r)] \tag{S4}$$

with B a constant to be determined from the boundary conditions. A particular solution can be obtained *via* the variation of constant, amounting to substituting $C_h(r)$ into Eq. (4) of the manuscript and treating B as a function of r, $B \to B(r)$. This substitution provides the following equation for $B(r)$

$$D\frac{dB(r)}{dr} = \frac{A}{r^2}\exp[-\phi(r)]$$

leading to the particular solution

$$C_p(r) = \frac{A}{D}\exp[\phi(r)] \int_{r_e}^{r} d\rho \, \frac{\exp[-\phi(\rho)]}{\rho^2} \; .$$

Now, combining $C_h(r)$ and $C_p(r)$, the general solution of Eq. (S1) reads

$$C(r) = \exp[\phi(r)]\left(B + \frac{A}{D}\int_{r_e}^{r} d\rho\, \frac{\exp[-\phi(\rho)]}{\rho^2}\right) . \tag{S5}$$

The constants A and B are determined by fulfilling the two boundary conditions, Eq. (S2) and $C(r_b) = C_0$. They turn to be

$$A = \frac{C_0 D e^{-\phi(r_b)}}{\dfrac{D}{k r_e^2} e^{-\phi(r_e)} + \displaystyle\int_{r_e}^{r_b} d\rho \frac{e^{-\phi(\rho)}}{\rho^2}} \tag{S6}$$

and

$$B = \frac{e^{-\phi(r_e)}}{k r_e^2} \frac{C_0 D e^{-\phi(r_b)}}{\dfrac{D}{k r_e^2} e^{-\phi(r_e)} + \displaystyle\int_{r_e}^{r_b} d\rho \frac{e^{-\phi(\rho)}}{\rho^2}} . \tag{S7}$$

These expressions, when plugged into Eq. (S5), yield the final result

$$C(r) = C_0 e^{\phi(r)-\phi(r_b)} \frac{\dfrac{D}{k r_e^2} e^{-\phi(r_e)} + \displaystyle\int_{r_e}^{r} d\rho \frac{e^{-\phi(\rho)}}{\rho^2}}{\dfrac{D}{k r_e^2} e^{-\phi(r_e)} + \displaystyle\int_{r_e}^{r_b} d\rho \frac{e^{-\phi(\rho)}}{\rho^2}} . \tag{S8}$$

Eq. (6) of the main text is obtained by assuming that $r_b \gg r_e$, (virtually $r_b \to \infty$) and considering $\phi(\infty) = 0$.

Moreover, from Eq. (S1), it is straightforward to see that the radial flux is $J(r) = -A/r^2$. The negative sign stems from the current direction which is always towards the origin where the pore entrance is located. However to compute the capture rate, we are interested in the absolute value of $J(r)$, thus

$$f = 2\pi r^2 J(r) = 2\pi A . \tag{S9}$$

As discussed in the text, we will employ the following model for $\phi(r)$ (see Eq. (17) of the main text)

$$\phi(r) = \frac{1}{D}\left(\frac{a_2}{r} + \frac{a_3}{2r^2}\right) , \tag{S10}$$

hence the integrals in Eqs. (S5)-(S7) can be expressed in terms of error functions, thus $C(r)$ takes on the explicit form

$$C(r) = C_0 e^{\phi(r)-\phi(r_b)} \frac{\dfrac{e^{-\phi(r_e)}}{kr_e^2} + \sqrt{\pi} g e^{(ga_2)^2} \left\{ \mathrm{erf}\left[g\left(a_2 + \dfrac{a_3}{r_e} \right) \right] - \mathrm{erf}\left[g\left(a_2 + \dfrac{a_3}{r} \right) \right] \right\}}{\dfrac{e^{-\phi(r_e)}}{kr_e^2} + \sqrt{\pi} g e^{(ga_2)^2} \left\{ \mathrm{erf}\left[g\left(a_2 + \dfrac{a_3}{r_e} \right) \right] - \mathrm{erf}\left[g\left(a_2 + \dfrac{a_3}{r_b} \right) \right] \right\}} \tag{S11}$$

where $g = 1/\sqrt{2Da_3}$. From Eqs.(S6) and (S9), we obtain the capture frequency when electrophoretic, advection and dielectrophoretic effects are concurrent

$$f = \left(\frac{1}{f_a} + \frac{1}{f_e} \right)^{-1} \tag{S12}$$

with

$$f_a = \frac{2\pi C_0 e^{-\phi(r_b)}}{\sqrt{\pi} g e^{(ga_2)^2} \left\{ \mathrm{erf}\left[g\left(a_2 + \dfrac{a_3}{r_e} \right) \right] - \mathrm{erf}\left[g\left(a_2 + \dfrac{a_3}{r_b} \right) \right] \right\}} \tag{S13}$$

and

$$f_e = 2\pi C_0 k r_e^2 \, e^{-\phi(r_b)+\phi(r_e)} \tag{S14}$$

the two contributions due to the transport of the particle from the bulk to the pore entrance (approach frequency f_a) and to the partial adsorbtion at the pore entrance (entrance frequency f_e). Notice that the formulas in the main text assume $r_b \to \infty$.

In the following of the appendix, we derive the explicit expression of $C(r)$ and Eq. (S12) when $a_3 = 0$ (only electrophoresis and advection are active) and when $a_2 = 0$ (only dielectrophoresis is active).

Electrophoresis and advection: $a_3 = 0$

This case corresponds to take the limit $a_3 \to 0$ in Eqs. (S11),(S13),(S14). This limit implies that $g \to \infty$, therefore we can use the asymptotic expansion of the error function, $\mathrm{erf}(s)$, for

large arguments,

$$\mathrm{erf}(s) \simeq \frac{1-\exp(-s^2)}{\sqrt{\pi}s}.$$

To see how the simplification works, we consider only the term with erf at the numerator of Eqs. (S11)

$$\sqrt{\pi}ge^{(ga_2)^2}\left\{\frac{e^{-g^2(a_2+a_3/r)^2}}{\sqrt{\pi}g(a_2+a_3/r)} - \frac{e^{-g^2(a_2+a_3/r_e)^2}}{\sqrt{\pi}g(a_2+a_3/r_e)}\right\}$$

then expanding the squares in the argument of the exponentials and taking $a_3 \to 0$, the term simplifies to

$$\frac{\exp[-a_2/(Dr)] - \exp[-a_2/(Dr_e)]}{a_2}.$$

Operating the same procedure to the entire Eq. (S11) yields stationary concentration under electrophoretic and advection effects

$$C(r) = C_0 e^{\gamma(1/r-1/r_b)} \frac{\dfrac{e^{-\gamma/r_e}}{kr_e^2} + \dfrac{e^{-\gamma/r} - e^{-\gamma/r_e}}{a_2}}{\dfrac{e^{-\gamma/r_e}}{kr_e^2} + \dfrac{e^{-\gamma/r_b} - e^{-\gamma/r_e}}{a_2}} \tag{S15}$$

where $\gamma = a_2/D$. Examples of $C(r)$ profiles at different values of k are shown in Fig. S1a. A similar computation on Eq. (S6) leads to the following expression for the approach and the entrance frequency

$$f_e = 2\pi C_0 k r_e^2 e^{-\gamma/r_b+\gamma/r_e} \tag{S16}$$

$$f_a = \frac{2\pi C_0 a_2 e^{-\gamma/r_b}}{e^{-\gamma/r_b} - e^{-\gamma/r_e}} \tag{S17}$$

that, for $r_b \to \infty$, reduces to Eq. (20) of the main text.

Pure dielectrophoretic effect: $a_2 = 0$

When $a_2 = 0$ in $\phi(r)$ and in Eq. (S11), we are considering a particle driven to the pore by a pure dielectrophoretic mechanism. The steady concentration for this process can be easily obtained from Eq. (S11) without difficulty. Examples of $C(r)$ profiles are reported in

Fig. S1b for different values of k. The corresponding approach and entrance frequency are

$$f_e = 2\pi C_0 k r_e^2 e^{-\gamma/r_b^2+\gamma/r_e^2} \ , \tag{S18}$$

$$f_a = \frac{2\pi C_0 e^{-\gamma/r_b^2}}{\sqrt{\pi} g \left\{ \mathrm{erf}\left[g\left(\frac{a_3}{r_e}\right)\right] - \mathrm{erf}\left[g\left(\frac{a_3}{r_b}\right)\right]\right\}} \ , \tag{S19}$$

where now $\gamma = a_3/D$. Also in this case, we assumed in the main text $r_b \gg r_e$, corresponding to $r_b \to \infty$ and obtaining, after a few manipulations, Eq. (21) of the main text.

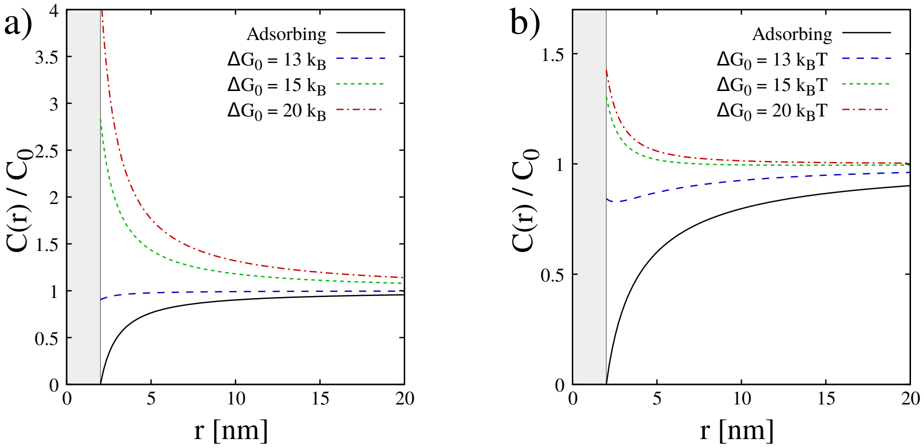

Figure S1: **Particle concentration for different entrance barriers.** The rescaled concentration profiles $C(r)/C_0$ refer to the same system of Fig. (5) of the main text. In panel a), $a_3 = 0$ (Eq. (S15), no dielectrophoresis), while, in panel b), $a_2 = 0$, (Eq. (S11), pure dielectrophoresis). In both panels, $\Delta V = 0.1V$ and $r_b \to \infty$. The gray areas correspond to the pore access ($0 \le r \le r_e$, with $r_e = 2$ nm). For these cases, when $\Delta G_0 \lesssim 10\ k_B T$, the concentration profile is practically indistinguishable from the perfectly adsorbing case ($k \to \infty$, $\Delta G_0 \to -\infty$). This in agreement with the capture frequency plots of Fig. (5) of the main text where it is apparent that at $\Delta V = 0.1$, $f \simeq f_a$, *i.e.* the process is in the transport limited regime.

Supplementary note S2: Estimation of the Electroosmotic flow

Here, for the reader's convenience, we report the standard argument to derive Eq. (22) of the main text which links the estimated electroosmotic flow $Q_f = G_{eo}\Delta V$ to the pore surface charge q_w. This expression is used in the solid state nanopore example of Fig. 3 and Fig. 4 of the manuscript. We employ the ideal electroosmotic flow formulation for a cylinder of radius r_p and length L, neglecting the entrance effect, an assumption that is valid for $L \gg r_p$. The cylinder surface is assumed to be uniformly charged with density q_w. The general formulation of the problem is within the Poisson-Nernst-Planck-Navier-Stokes (PNP-NS) approach.[1] If the wall is made of an insulating material, the surface charge q_w is related to the component of the electric field normal to the surface, E_n, as

$$E_n = \frac{q_w}{\epsilon_0 \epsilon_e} \ , \tag{S20}$$

with ϵ_e the relative permittivity of the electrolyte solution. Eq. (S20), together with a no-slip boundary condition (or partial slip, if appropriate) constitutes the boundary condition for the PNP-NS system. In general, NS-PNP system requires numerical solvers. Nevertheless, for an infinite circular cylinder, no-slip wall, and external electrical field ($E_e = -\Delta V/L$) acting along the pore axes, an analytical solution in terms of Bessel function can be found, see[1] among others. The resulting velocity profile is zero at the pore wall (no slip) and it increases in a shell whose thickness is the Debye length λ_D until, if the pore is large enough ($r_p \gg \lambda_D$), it reaches the plateaux value,

$$u_{eo} = \frac{q_w \lambda_D}{\rho \nu} \frac{\Delta V}{L} \ , \tag{S21}$$

with ρ and ν the density and the kinematic viscosity of the electrolyte solution. Note that usually this expression is written in terms of the dynamic viscosity $\mu = \rho\nu$, however, here,

since the symbol μ was already used for particle mobility, we prefer to use the kinematic viscosity ν. An estimation of the volumetric flow rate is, thus, $Q_f = \pi r_p^2 u_{eo}$, that leads to the definition of the electroosmotic conductance, G_{eo}, reported in Eq. (22) of the manuscript. The minus in Eq. (22) of the manuscript stems from the convection made on the ground electrode. The interested reader may find a model for entrance effects and a discussion on the validity of the theory in Ref.[2]

Supplementary note S3: Local increase of E due to blockade

Here we report a toy model explaining the increase of the electric field intensity E, when a molecule partially blocks the pore. We consider a cylindrical pore of length L and section A partially and co-axially occupied by a cylindrical molecule of section A_m. Let L_1 denote the length of the portion of the molecule inside the pore, see Fig S2. Using a quasi-1D model for the estimation of the pore resistance R, we get

$$R = R_1 + R_2 = \frac{L_1}{A_1 \sigma} + \frac{L_2}{A_2 \sigma} \tag{S22}$$

with σ the electrolyte conducibility, the subscript 1 refers to the portion of the pore occupied by the molecule, while 2 corresponds to the free region. With reference to Fig. S2, $A_1 = A - A_m$ is the pore section available to the ion passage in the region occupied by the molecule while $A_2 = A$ is the free pore section and $L_2 = L - L_1$ the length of the portion of the pore not occupied by the molecule. For $L_1 = 0$ (free pore), Eq. (S22) gives the usual expression for a cylindrical pore, $R = L/(A\sigma)$.

Our aim is to calculate the ratio, $s = E_1/E_0$, used in the analysis of the dielectophoresis experiment reported in Fig. 7i of the manuscript and in the discussion of the Larkin *et al.*[3] results, where E_1 is the electrical field intensity in the region 1, when the molecule partially blocks the ion flow and the subscript 0 refers to the completely free pore. At constant applied voltage ΔV, the increase in the system resistance R is associated to a reduction of the current $I = \Delta V/R$. I is the same in any section of the pore, so, the current flux reads $J(z) = I/A(z)$ with z denoting a coordinate along the pore axis and $A(z)$ the pore section. Finally, using the Ohm law $J = \sigma E$, we get

$$s = \frac{E_1}{E_0} = \frac{J_1}{J_0} = \frac{1}{L_1/L + (1 - L_1/L)(A_1/A)} \, . \tag{S23}$$

Fig. S2b shows s as a function of L_1/L for different values of A_1/A. The maximum value occurs for $L_1 \to 0$ (molecule at the pore entrance) where $s \to A/A_1$. Then, as the molecule penetrates the pore, s decreases monotonically to the value $s = 1$, attained for $L_1 \to L$ (pore fully occupied by the molecule) where $E_1 = E_0$. This possibility occurs only if the molecule is longer than the pore. As a final note, if $A_m = A$, $A_1 = 0$, hence, no ion current sets in. Consequently, electrical field needs to be determined *via* electrostatic and not using Ohm law as done here.

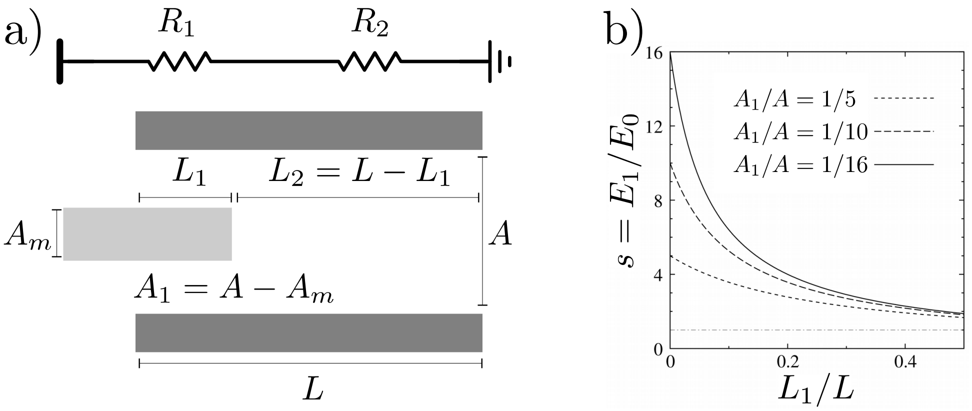

Figure S2: **Increase of electrical field during capture.** When a molecule enters the pore, it reduces the room available for ion passages. This results in a local increase of the electric field. Panel a) sketches the toy model, panel b) reports the increase of the electric field intensity, $s = E_1/E_0$, in the region occupied by the molecule (E_1) with respect to the case where the pore is completely free (E_0).

Supplementary note S4: SV28 peptide structure prediction

Here we report the details on the computational prediction of the structure of peptide SV28, whose sequence is RGSYSVSVSVSYDSDGSYSVSVSVSYGR. To predict the SV28 structure we used the PEP-FOLD3.5 server.[4] All the best 5 models provided by the server are $\beta-$hairpins. For each model, we calculated the distance d between the center of mass of the two Arginines (positively charged) and the center of mass of the two Aspartic acids (negatively charged), finding very small variations among the 5 models, $d \in (3.85 - 4.14)$ nm. As a further confirmation, we used VMD[5] to calculate the electric dipole moment $\mathbf{p}$ of the predicted structures, obtaining $|\mathbf{p}| \simeq 8.3\ e \cdot \text{nm}$. This value is very close to the one used in our dumbbell model, that amounts to $|\mathbf{p}| = 2ed = 8\ e \cdot \text{nm}$.

References

1. Bruus, H. *Theoretical Microfluidics*; Oxford university press, Oxford UK, 2008; Vol. 18.

2. Sherwood, J. D.; Mao, M.; Ghosal, S. Electroosmosis in a Finite Cylindrical Pore: Simple Models of End Effects. *Langmuir* **2014**, *30*, 9261–9272.

3. Larkin, J.; Henley, R. Y.; Muthukumar, M.; Rosenstein, J. K.; Wanunu, M. High-Bandwidth Protein Analysis Using Solid-State Nanopores. *Biophys. J.* **2014**, *106*, 696–704.

4. Lamiable, A.; Thévenet, P.; Rey, J.; Vavrusa, M.; Derreumaux, P.; Tufféry, P. PEP-FOLD3: Faster de Novo Structure Prediction for Linear Peptides in Solution and in Complex. *Nucleic Acids Res.* **2016**, *44*, W449–W454.

5. Humphrey, W.; Dalke, A.; Schulten, K. VMD: Visual Molecular Dynamics. *J. Mol. Graph.* **1996**, *14*, 33–38.
